# Thermoresponsive Bacteriophage Nanocarrier as a Gene Delivery Vector Targeted to the Gastrointestinal Tract

**DOI:** 10.1016/j.omtn.2018.04.012

**Published:** 2018-05-01

**Authors:** Katawut Namdee, Mattaka Khongkow, Suwimon Boonrungsiman, Naiyaphat Nittayasut, Paladd Asavarut, Sasithon Temisak, Nattika Saengkrit, Satit Puttipipatkhachorn, Amin Hajitou, Kiat Ruxrungtham, Teerapong Yata

**Affiliations:** 1 National Nanotechnology Centre (NANOTEC), National Science and Technology Development Agency, Pathumthani, Thailand; 2 Cancer Phage Therapy Group, Division of Brain Sciences, Imperial College London, London, UK; 3 Bio Analysis Group, Chemical Metrology and Biometry Department, National Institute of Metrology (NIMT), Pathumthani, Thailand; 4 Department of Manufacturing Pharmacy, Faculty of Pharmacy, Mahidol University, Bangkok, Thailand; 5 Vaccine and Cellular Immunology Laboratory, Vaccine Research Center (ChulaVRC) and Department of Medicine, Faculty of Medicine, Chulalongkorn University, Bangkok, Thailand

**Keywords:** bacteriophage, thermoresponsive, DNA, gene, gastrointestinal tract, nanocarrier

## Abstract

The use of the gastrointestinal tract as a site for the local delivery of DNA is an exciting prospect. In order to obtain an effective vector capable of delivering a gene of interest to target cells to achieve sufficient and sustained transgene expression, with minimal toxicity, we developed a new generation of filamentous bacteriophage. This particular bacteriophage was genetically engineered to display an arginine-glycine-aspartic acid (RGD) motif (an integrin-binding peptide) on the major coat protein pVIII and carry a mammalian DNA cassette. One unanticipated observation is the thermoresponsive behavior of engineered bacteriophage. This finding has led us to simplify the isolation method to purify bacteriophage particles from cell culture supernatant by low-temperature precipitation. Our results showed that, in contrast to non-surface modified, the RGD-modified bacteriophage was successfully used to deliver a transgene to mammalian cells. Our *in vitro* model of the human intestinal follicle-associated epithelium also demonstrated that bacteriophage particles were stable in simulated gastrointestinal fluids and able to cross the human intestinal barrier. In addition, we confirmed an adjuvant property of the engineered bacteriophage to induce nitric oxide production by macrophages. In conclusion, our study demonstrated the possibility of using bacteriophage for gene transfer in the gastrointestinal tract.

## Graphical Abstract

**Figure:**
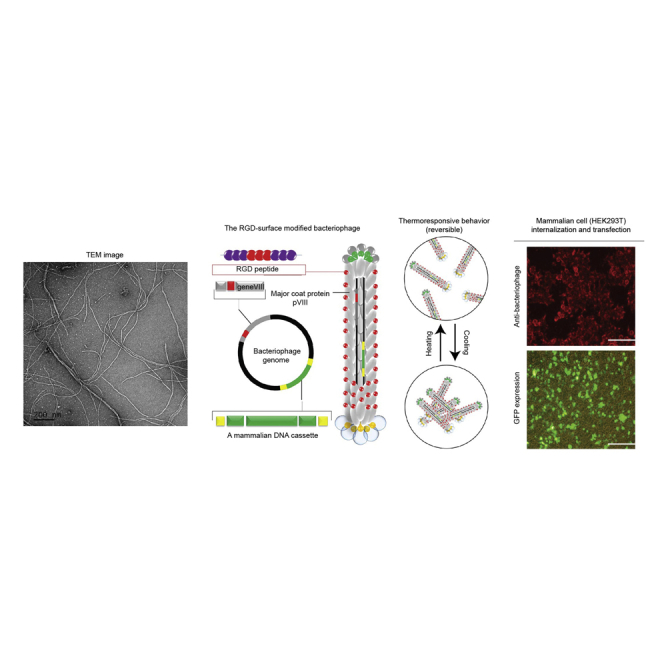

## Introduction

Gene transfer is the introduction of nucleic acids into cells. This technology provides the ability to genetically manipulate the target cells and has contributed to various applications.^1^ An important approach in the field of cell and molecular biology is the transfer of genes of interest in selected cell cultures or animal models, with the aim to study the gene’s function.^2^ Moreover, the production of therapeutic proteins, such as hormones, growth factors, cytokines, and immunoglobulins, that is used to treat a broad spectrum of diseases largely relies on the expression of recombinant proteins in cultivated mammalian cells.^3^ One of the most important contribution of gene transfer innovation involves the direct use of DNA as a vaccine to prevent diseases. This so-called “DNA vaccination” holds the promise for preventing a large number of infectious diseases.^4^

DNA vaccination, also referred to as genetic immunization, involves the introduction of a DNA molecule, generally a circular plasmid with an antigen-encoding gene, into a host where the expression of an antigenic protein in host cells can elicit both humoral and cellular immune responses.^5^ DNA vaccines offer significant advantages over traditional attenuated, killed, or subunit vaccines. They are cheaper and easier to produce than recombinant protein vaccines and do not require complete knowledge of the pathogen. They are stable at room temperature and therefore easy to transport and store.^4^

Most commercial vaccines available today are delivered by injection, intramuscular or subcutaneous routes. Recently, needle-free vaccine delivery has received considerable attention.^6^, ^7^, ^8^ Orally administered vaccine is of particular interest due to its easy handling, low cost of production, and induction of mucosal immunity.^9^ The development of safe and cheap carriers capable of efficient, selective, and targeted delivery of DNA vaccines to target cells is of great importance.

Recently, a number of investigations have focused on designing bio-inspired nanocarriers, such as bacteriophage.^10^ Filamentous bacteriophages, a novel class of nanomaterials, have been exploited as a vaccine delivery vector.^11^ A vast majority of previous reports have utilized filamentous bacteriophages as immunogen carriers for boosting immune response against peptides or proteins displayed on their surface.^11^ Bacteriophages have several advantages that make them well suited for developing vaccine delivery platforms. Phage itself serves as a strong adjuvant and thus promotes excellent immunity against antigens being delivered.^12^, ^13^ Additionally, unmethylated CpG motifs of the phage genome act as immunostimulants, which further enhances immunity.^14^ It has been demonstrated that bacteriophage-based particles can be internalized in associated cells, successfully digested, and targeted both to major histocompatibility complex (MHC) class I and class II antigen-processing pathways.^15^ The natural stability of phage particles makes them easy and cheap to process and purify by simple centrifugation steps. Importantly, they are very thermostable and resistant to degradation, making them ideally suited for shipping, storage, and delivery without requiring refrigeration.^16^

In this study, we used filamentous bacteriophage to construct an ideal vehicle for delivering DNA vaccine by oral route. In particular, we genetically engineered (1) the bacteriophage genome to carry a mammalian DNA cassette encoding a protein or antigen of interest and (2) the major coat protein pVIII to express at its N terminus the *arginine*-glycine-aspartic acid (RGD) motif (an integrin-binding peptide) able to target bacteriophage particles to appropriate cells in the gastrointestinal tract.

## Results

### Construction and Characterization of RGD-Surface-Modified Bacteriophage

**Figure 1:**
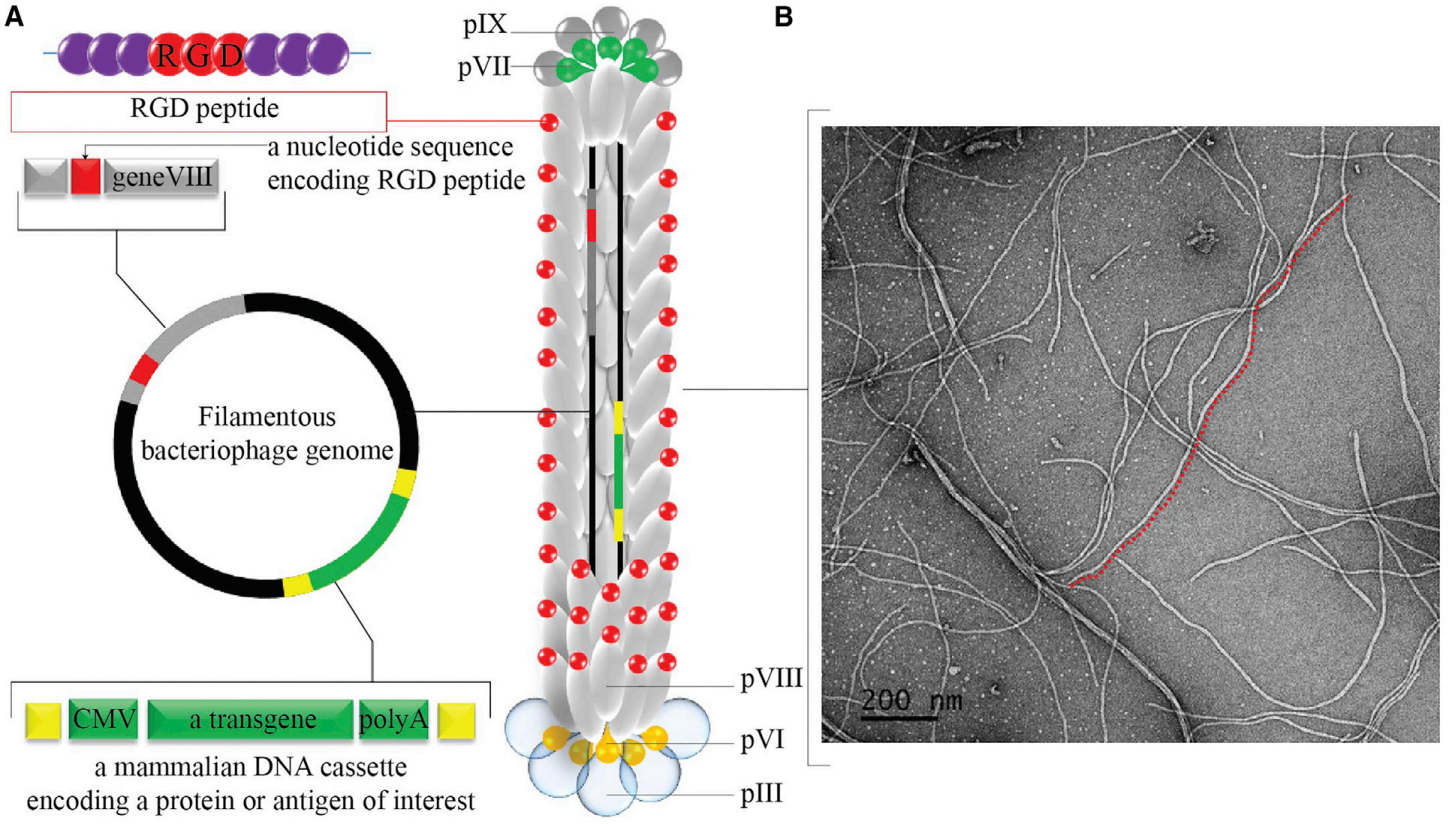
Design and Characterization of the RGD-Surface-Modified Bacteriophage Nanocarrier (A) Schematic representation of the RGD-surface-modified bacteriophage nanocarrier. The vector displays the RGD peptide on the pVIII major coat protein and a protein-encoding gene cassette inserted in the bacteriophage genome. (B) Morphological characterization of RGD-surface-modified bacteriophage nanocarrier by transmission electron microscopy is shown.

We generated the RGD-bacteriophage by introducing the integrin-binding (RGD) peptide to the N terminus of each copy of the major coat protein pVIII as schematically shown in Figure 1A. To engineer this new bacteriophage, we used the modified fUSE5-MCS vector, in which the multiple cloning site has been inserted into the intergenic region of fUSE5 vector as previously described.^17^ The first genetic engineering step was to introduce the RGD peptide between residues Gly3 and Asp4 of the N-terminal region of the major coat protein pVIII using site-directed mutagenesis. The correct insertion of the RGD nucleotide sequence of the resultant vector was confirmed by sequencing analysis. Next, the transgene cassette (GFP reporter gene) was inserted into the multiple cloning site of the engineered vector. Finally, a positive clone was used for bacteriophage propagation followed by purification (see Materials and Methods).

RGD-surface-modified bacteriophages were first characterized by negative staining and analysis by transmission electron microscopy. As shown in Figure 1B, engineered bacteriophage particles are flexible and slender cylindrical filaments less than 10 nm in diameter and approximately 1,400 nm in length.

### Physicochemical and Biological Characterization of Bacteriophage Nanocarriers

Next, we investigated the charge characteristic of bacteriophage particles by measuring their ζ potential. Similar to the non-surface-modified (NS) bacteriophage, the results show the negative charge of the RGD-bacteriophage at physiological pH (Table 1).

**Table 1:** Physical Characterization of Bacteriophage Nanocarriers

Samples	ζ Potential (mV)
NS	−18.13 ± 0.29
RGD	−19.53 ± 0.58

**Figure 2:**
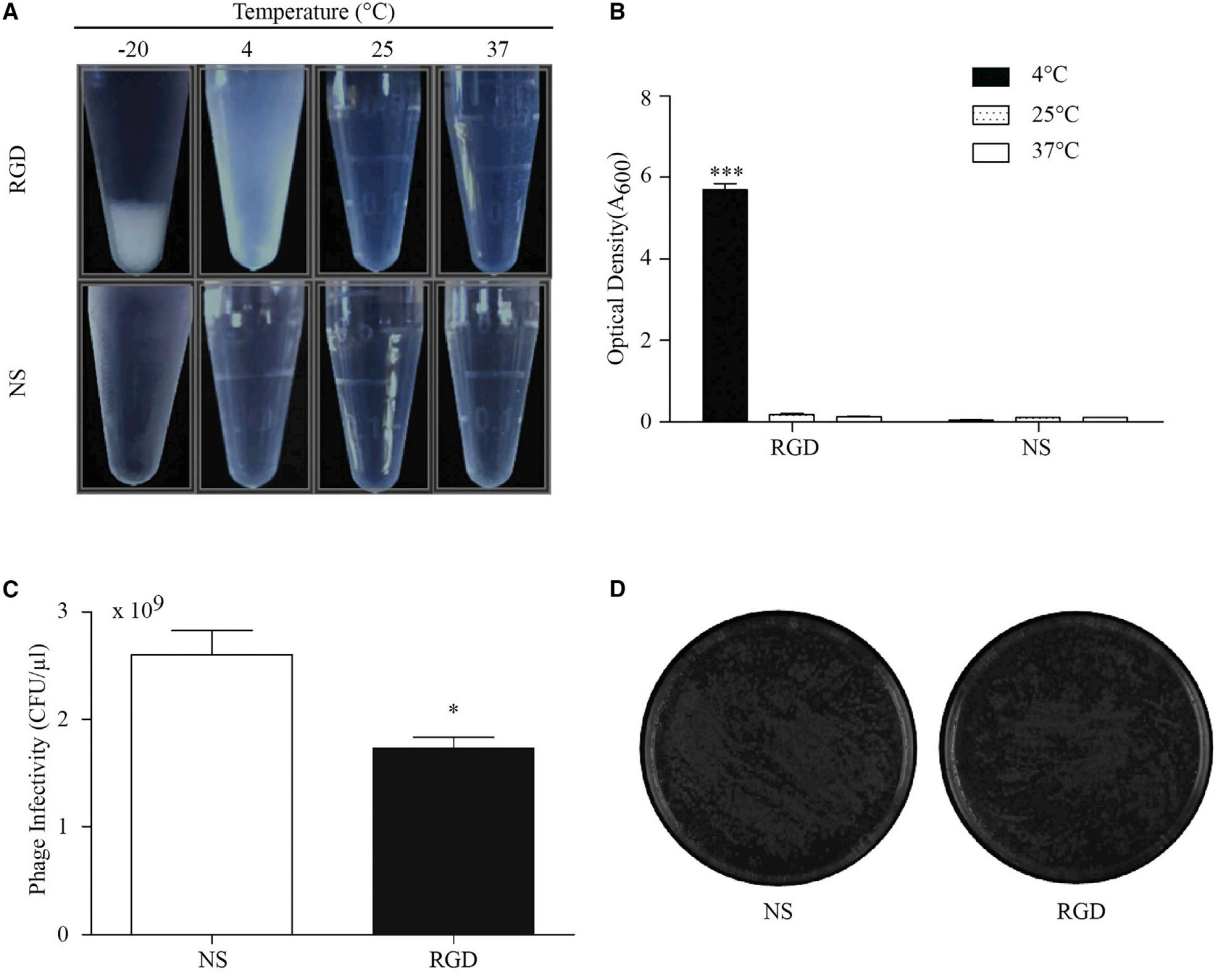
Physicochemical and Biological Properties of Bacteriophage Nanocarriers (A) Thermoresponsive behavior of the RGD- and NS-bacteriophage in PBS (pH 7.4) at different temperatures. (B) Optical density of the RGD- and NS-bacteriophage at different temperatures is shown. (C) Infectivity (colony forming units/μL) was calculated from the number of colonies that grew on the plates overnight. (D) LB-agar plates showing the colony formation between the NS- and the RGD-bacteriophage are shown. One representative plate of each bacteriophage is shown. Data represent the mean + SEM of triplicate samples from one representative experiments of three, significant difference; n.s., not significant; *p < 0.05; ***p < 0.001 (two-way ANOVA).

One unanticipated finding was thermoresponsive aggregation behavior of bacteriophage nanocarriers. The RGD-bacteriophage was studied according to its reversible thermoresponsive aggregation behavior in aqueous solution as shown in Figure 2A. The RGD-bacteriophage, but not NS-bacteriophage, spontaneously precipitated from water upon cooling. Upon increasing the temperature to 25°C and 37°C, dissolution of the aggregates could be clearly observed, and the solution of the RGD-bacteriophage became transparent instantly. This kind of behavior was not observed for the NS-bacteriophage. Also, turbidity measurements (Figure 2B) revealed significant differences in the solution behavior of the RGD- and the NS-bacteriophage. A 50-mg/mL solution of the RGD-bacteriophage appeared to be turbid (optical density at 600 nm [OD_600_] = 6) at 4°C, indicating the presence of large aggregates. Upon increasing the temperature to 25°C and 37°C, optical density dropped sharply to 0.

We also evaluated the effect of coat protein mutation on the infectivity of engineered bacteriophage vectors. Bacteriophage concentration was measured by spectrophotometric quantification of DNA and protein. After being adjusted to an identical bacteriophage particle number as previously described,^18^ clones were assayed for infectivity by counting colonies (colony-forming units [CFUs]) formed on Luria-Bertani (LB) broth agar plates. There was a difference in their ability to infect *E. coli* bacteria compared to the NS-bacteriophage, as shown in Figure 2C. The RGD-bacteriophage yielded lower number of colonies (Figure 2D). This suggests that the insertion of a RGD sequence on wild-type pVIII can affect its ability to infect *E. coli* host cells.

### Cell Surface α_v_ Integrin Receptors Binding and Transgene Delivery Characteristics of the Engineered Bacteriophage Nanocarrier

**Figure 3:**
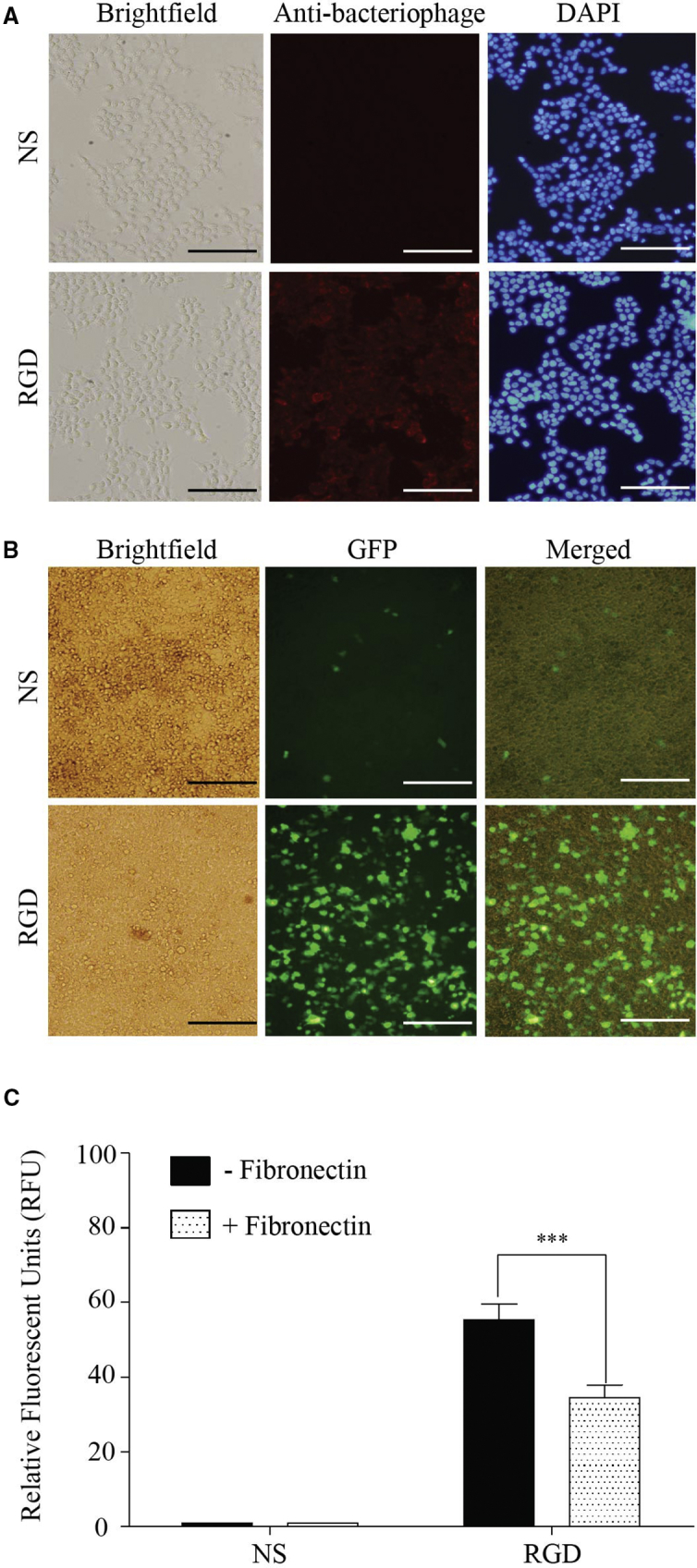
Evaluation of the Targeting of Mammalian Cells by the Engineered Bacteriophage Nanocarrier (A) Immunofluorescence-based bacteriophage binding assay. Cultured HEK293T cells were incubated with the RGD- or NS-bacteriophage. The red color represents fluorescence from bacteriophage staining, and the blue color shows fluorescence of DAPI-stained cell nuclei. The scale bars represent 100 μm. (B) GFP expression observed after transfection of HEK293T cells with the RGD- or NS-bacteriophage is shown. The scale bars represent 100 μm. (C) Quantitative analysis of GFP level in the presence or absence of fibronectin is shown. Experiments were performed in triplicate and data presented as percentage of the mean of relative fluorescence units (RFU) of treated cells compared with the control HEK293T cells stably expressing GFP. Significant difference: n.s., not significant, ***p < 0.001

We validated the function of the RGD-targeting ligand displayed on the pVIII major coat protein by assessing binding to cells expression of integrin receptors. Immunofluorescence using antibodies against the bacteriophage coat protein was performed on highly integrin-expressing HEK293T cells.^19^ As shown in Figure 3A, we demonstrated targeting capabilities of the RGD-bacteriophage, indicating that the display of RGD peptide is functional. The NS-bacteriophage showed background signal only.

To examine that the RGD-bacteriophage can deliver transgenes into mammalian cells, we also carried out cell transfection experiments on HEK293 cells. Analysis of GFP expression showed GFP expression in cells transfected with the RGD-bacteriophage (Figure 3B). Low GFP expression was observed in the NS-bacteriophage-transfected cells (Figure 3B). The data prove that the RGD-bacteriophage successfully mediates transgene expression in mammalian cells superior to the NS-bacteriophage. We also investigated the effect of fibronectin (RGD motif-containing proteins) on the transfection efficiency of RGD-surface-modified bacteriophage. As anticipated, pretreatment of HEK293 cells with 0.2 mg/mL of fibronectin significantly decreased GFP transgene expression without significant signs of cytotoxicity, with an approximately 30% decrease (Figure 3C).

### The Stability of Engineered Bacteriophage at Different Acid pH and Enzymatic Fluids

**Figure 4:**
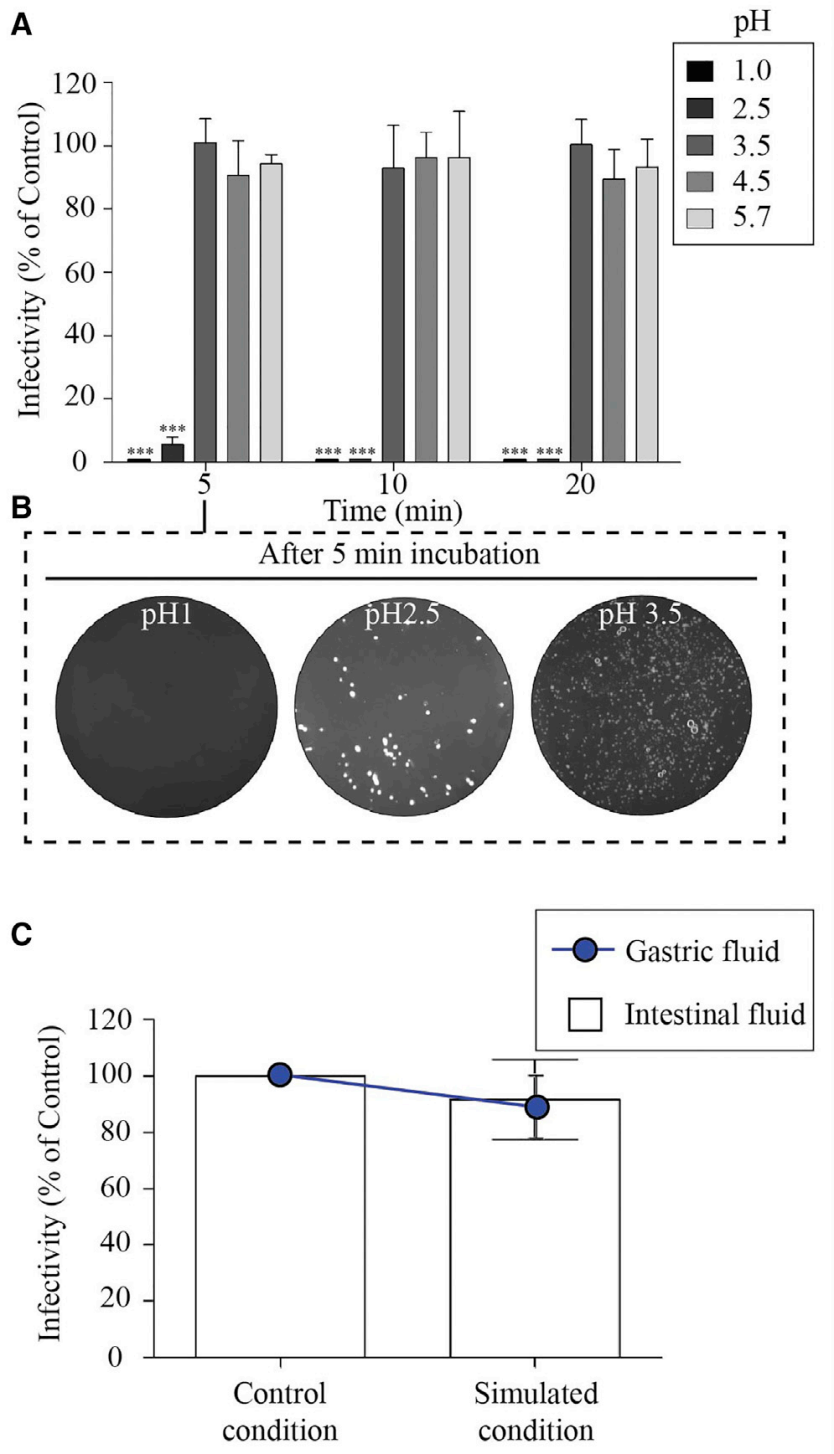
The Stability of Engineered Bacteriophage at Different Acid pH and Enzymatic Fluids (A) The effect of low pH on the survival of RGD-surface-modified bacteriophage. (B) LB-agar plates showing the colony formation of RGD-bacteriophage at different acid pH are shown. One representative plate of each bacteriophage is shown. (C) Stability of RGD-surface-modified bacteriophage under simulated gastric or intestinal conditions. Each measurement was performed in triplicate, and each experiment was repeated at least three times. The results are presented in mean infectivity (% of control) ± SEM. Significant difference: n.s., not significant, ***p < 0.001

The effect of low pH in the range 1.0∼5.7 on the survival of RGD-surface-modified bacteriophage is shown in Figures 4A and 4B. It was found that exposure to pH 3.5, 4.5, or 5.7 did not result in a significant reduction in infective titer over the course of 20 min. At pH 1.0, all bacteriophages were inactivated within 5 min. As shown in Figure 4C, the stability of engineered bacteriophage was also evaluated under simulated gastric and pancreatic conditions. Our results showed that RGD-surface-modified bacteriophage remained mostly unaffected in SGF after 1 hr of incubation. Similarly, exposure to pancreatic enzymes (Figure 4C) had no major effect on the viability of engineered bacteriophage after 120 min of incubation.

### Targeting of Gene Delivery to Intestinal Cells by the Engineered Bacteriophage Nanocarrier

**Figure 5:**
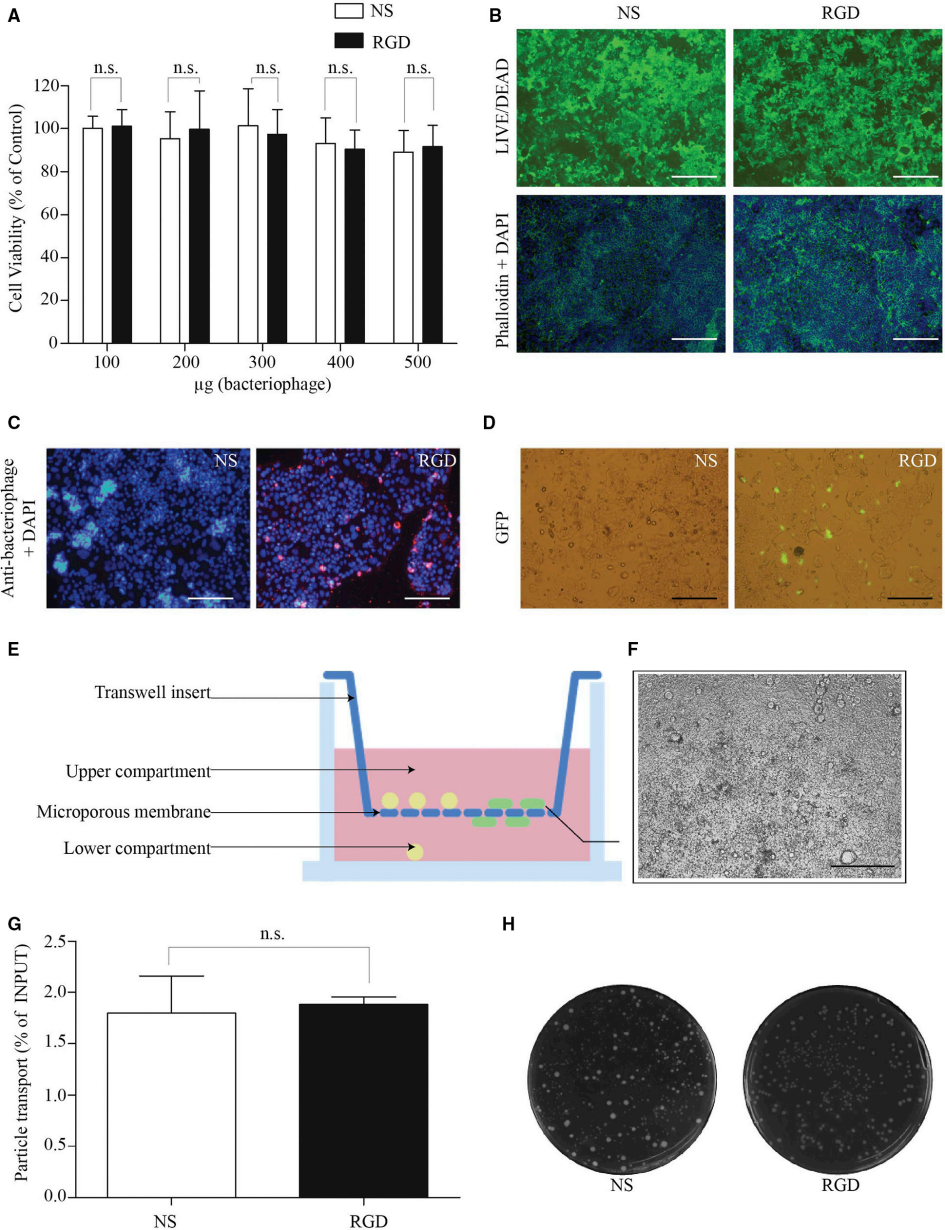
Targeting of Gene Delivery to Intestinal Cells by the Engineered Bacteriophage Nanocarrier (A) Cytotoxicity of bacteriophage nanocarriers at various concentrations in caco-2 cells. Cells were treated with increasing doses (μg/well) of the RGD- or NS-bacteriophage. Following 24 hr incubation, cell viability was determined by the CellTiter-Glo cell viability assay. (B) Morphological characteristics of Caco-2 cells visualized under the fluorescence microscope are shown. Cells were also stained with the reagents in the LIVE/DEAD Cell Viability/Cytotoxicity Assay Kit, Alexa-Fluor-584-conjugated phalloidin, and DAPI and visualized under the fluorescence microscope. The scale bars represent 100 μm. (C) Fluorescent microscopy of Caco-2 cells after treated with the RGD- or NS-bacteriophage is shown. Cells were immunofluorescently stained for bacteriophage particles (red). The scale bars represent 100 μm. (D) Analysis of transfection efficiency mediated by the engineered bacteriophage nanocarrier in caco-2 cells is shown. GFP expression was observed after transfection of caco-2 cells with the RGD- or NS-bacteriophage. The scale bars represent 100 μm. (E) Experimental setting to study the bacteriophage translocation across the cell layer by using caco-2/Raji B co-culture system is shown. Cells were co-cultured on Transwell filters before the addition of bacteriophage particles to the apical compartment. Later, the lower compartment was collected and processed for bacteriophage quantification. (F) The cell layer of caco-2/Raji B intestinal cell co-culture system is shown. The scale bar represents 100 μm. (G) Total bacteriophage titer translocated across the human intestinal follicle-associated epithelium is shown. The bacteriophage population transported through the cell layer was quantified using the *E. coli* infection method and counting colony-forming units. Data represent the mean + SEM of triplicate samples from one representative experiments of three, significant difference; *p < 0.05; **p < 0.01; ***p < 0.001 (t test). (H) LB-agar plates showing colony formation between the NS-bacteriophage and the RGD-bacteriophage are shown. One representative plate of each bacteriophage is shown.

We first studied cell viability, tight junction protein (F-actin) distribution, and the presence of normal nuclei in the Caco-2 cell line. No cytotoxicity was observed in the concentration ranges of the NS- or RGD-bacteriophage tested (Figure 5A). Figure 5B shows normal morphology of viable cells (green fluorescence) treated with the same concentration of the NS- or RGD-bacteriophage. Cells were also treated with Alexa-Fluor-584-conjugated phalloidin and DAPI to examine actin filaments and cell nuclei, respectively (Figure 5B). Phalloidin staining did not reveal alterations in the actin cytoskeleton in treatments with the RGD-bacteriophage and in cells treated with the NS-bacteriophage. Cell nuclei showed no signs of condensation or fractionation, again indicating no cytotoxicity or cell death.

Next, we evaluated the binding capacity of engineered bacteriophage by using caco-2 cells known to express high levels of integrin receptors.^20^ Fluorescence microscopy revealed that a large number of RGD-bacteriophages can bind to cell surfaces, whereas no bacteriophage particles were observed on cell surfaces incubated with the NS-bacteriophage (Figure 5C).

Then, we investigated the RGD-bacteriophage-mediated gene delivery to caco-2 cells. At day 5 post-transfection, representative microscopic imaging of caco-2, as shown in Figure 5D, revealed GFP expression in cells transfected with the RGD-bacteriophage compared with the NS-bacteriophage. No GFP expression was observed in cells treated with the NS-bacteriophage. These data confirm that the introduction of RGD peptide resulted in the RGD-bacteriophage that significantly boosted gene delivery to human intestinal cells.

We *next* assessed the ability of engineered bacteriophage to translocate across the cell layer by using *in vitro* model of the human intestinal follicle-associated epithelium.

The RGD- or NS-bacteriophage was applied to *in vitro* co-culture (as schematically shown in Figure 5E) and measured each titer of bacteriophage translocated across the cell layer (Figure 5F). As shown in Figures 5G and 5H, the RGD-bacteriophage revealed similar transcytosis efficacy compared with the NS-bacteriophage having no RGD peptide insert.

### NO Production by Macrophage Stimulated with Engineered Bacteriophage Nanocarriers

**Figure 6:**
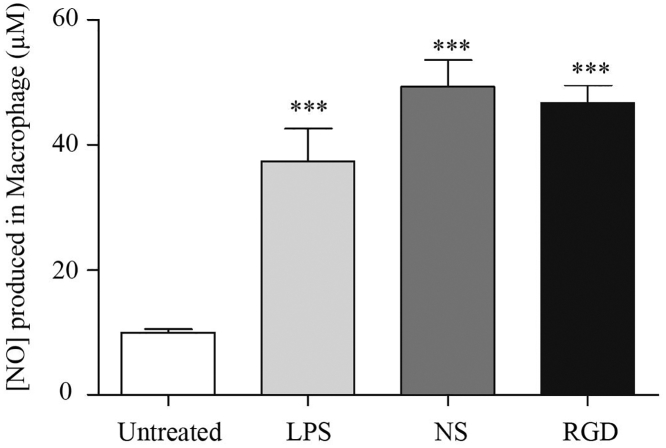
Nitric Oxide Production in RAW264.7 Cells Incubated with Bacteriophage Nanocarriers Lipopolysaccharide (LPS), a component from the outer membrane of gram-negative bacteria, was used as a positive control. Data are presented as the mean + SEM from replicates and are from at least three independent experiments (one-way ANOVA with Tukey’s post hoc test; ***p < 0.001).

In the present study, nitric oxide (NO) was used as an intercellular mediator produced in RAW264.7 cells in order to determine the adjuvant property of bacteriophage nanocarriers. After a 24-hr incubation, unstimulated macrophages secrete a background level of nitrite of about 2.8 μM in the culture medium (Figure 6). After treatment with lipopolysaccharide (LPS) (100 ng/mL) for 24 hr, nitrite concentration in the supernatant increased remarkably by about 43.1 μM. Interestingly, when cells were incubated with the RGD- or NS-bacteriophage, the amount of nitrite in the medium was similar to that in the LPS-treated cells, as shown in Figure 6. This finding suggests that activation of Raw264.7 cells by the bacteriophage can cause NO production.

## Discussion

One approach to targeted DNA delivery takes advantage of elevated levels of receptor expression in the appropriate cells.^21^ For example, the integrin receptor, which serves as a receptor for RGD-containing protein, has been found to be expressed on epithelium of small intestine,^22^ antigen-presenting cells of the immune system^23^ or dendritic cells,^24^ and microfold (M) cell of intestinal epithelium.^25^ A number of previous studies have demonstrated that RGD-labeled nanovaccines particularly concentrated in the mucosal membrane of the gastrointestinal tract, thus making it a promising strategy for oral vaccination.^26^, ^27^, ^28^, ^29^

Major (pVIII) coat proteins of bacteriophage provide the ability to display a wide range of foreign peptides of differing sizes. Therefore, it is possible to design novel bacteriophage constructs depending on which foreign proteins need to be displayed on the outer capsid. In this study, we proved the possibility of constructing bacteriophage-based nanocarrier, showing that moieties displayed on the bacteriophage capsid remain intact and functional by displaying the RGD ligand and carrying a mammalian DNA cassette expressing the reporter gene. We demonstrated that the presence of the RGD sequence on the coat protein of the engineered bacteriophage mediated a stronger uptake of these nanocarriers and gene expression in targeted cells expressing the integrin receptor.

The resulting particles were observed to have similar morphology to that of wild-type filamentous bacteriophage as previously characterized,^30^, ^31^ with the exception that particle length was measured at 1,400 nm long in comparison with 1,000 nm reported for the wild-type viruses. This is consistent with previous observations that the filamentous bacteriophage-derived particles can grow in length to accommodate the size of the genomic DNA it packages.^11^

Although the display of the RGD peptide on pVIII protein of engineered bacteriophage slightly affected their ability to infect *E. coli* bacteria, the yield of the engineered bacteriophage was considered high in titer and is within a workable range. Our finding is in agreement with a previous study showing that the display of peptides on wild-type pVIII affects their ability to infect bacteria.^32^

Another unexpected advantage of the RGD-surface-modified bacteriophage is spontaneous precipitation at low temperature. This kind of behavior was not observed in the non-surface-modified bacteriophage. In fact, the standard method to isolate and purify bacteriophage particles from the supernatant of bacteria host cell culture is the use of polyethylene glycol (PEG)/NaCl solution for precipitation.^33^, ^34^, ^35^ Having shown that lowering the temperature causes the precipitation of the RGD-bacteriophage solution, the separation of particles from the cell culture supernatant could be carried out without the addition of PEG and NaCl solution. This finding has led us to simplify the isolation method to purify bacteriophage particles from cell culture supernatant by centrifugation at low temperature. This simple and economical protocol for bacteriophage isolation is well suited for large-scale production.

As aforementioned, oral DNA vaccine delivery systems must be capable of delivering DNA vaccine to appropriate cells. DNA vaccine vectors could adhere to gastrointestinal epithelia, be transported across the intestinal barrier by M cells, and transfect epithelial and/or immune cells in the gut-associated lymphoid tissues (GALT), a specialized tissue that comprises the majority of T cells and a significant B cell compartmen,^36^, ^37^ either directly or through antigen transfer.^38^ Using the host cellular machinery, the DNA vaccine enters the nucleus of transfected cells and initiates production of the antigen protein. As proof of concept, the engineered bacteriophage that displays RGD peptide could be effectively internalized by integrin-expressing cells. Most importantly, the RGD-surface-modified bacteriophage can mediate transgene expression in integrin-expressing cells superior to the non-surface-modified bacteriophage. The competition experiment also confirms that gene delivery by the RGD-surface-engineered bacteriophage is targeted, specific and dependent on α_v_ integrin receptors.

Orally administered bacteriophage must tolerate the passage through the gastrointestinal tract. Bacteriophage can become irreversibly damaged by exposure to low pH values,^39^ a condition typically found in the gastrointestinal tract of animals. Although RGD-surface-modified bacteriophages remained unaffected at pH above 3, they were found to be extremely sensitive to highly acidic environments and were destroyed after a 5-min exposure to pH below 2. This observation is consistent with a previous study.^40^ Indeed, filamentous bacteriophages were considered to be more resistant to lower pH as compared to other types of bacteriophages. For example, T4 bacteriophages were inactivated at pH 4 (M. Kłak et al., 2010, First International Congress for Viruses of Microbes). T1 and PM2 bacteriophage particles were inactivated at pH 3.0 and 5.0, respectively.^39^

*In vitro* studies were also used to determine the effects of gastric and intestinal fluids on their viability. Engineered bacteriophages were found to be resistant to hydrolytic enzymes (pepsin), lipase, amylase, and protease (pancreatin). Therefore, enzymatic degradation does not appear to be a major concern for the oral delivery of engineered bacteriophage, with less than 10% reduction in the bacteriophage titer.

However, these problems could be addressed by encapsulation of bacteriophages in order to increase their stability and survival in harsh environments. There is a large volume of published studies describing the formulation and encapsulation of bacteriophage for improved stability and survival of bacteriophage in micro- and nanostructured materials.^41^

We observed no apparent advantage to using non-surface-modified or RGD-surface-modified bacteriophage to improve the transport across the *in vitro* human intestinal barrier. A possible explanation for our observation in the present study is the nature of bacteriophages and their ability to cross intestinal barrier. In fact, bacteriophages can pass the intestinal wall and migrate to GALT.^36^, ^37^

We also investigated whether the engineered bacteriophage nanocarrier was toxic against the human intestine. Our results suggested that the engineered bacteriophage is safe and biocompatible. In fact, bacteriophages have long been administered to humans; for example, its therapeutic use to treat pathogenic bacterial infection,^42^ and certain bacteriophage preparations have recently been approved by the US Food and Drug Administration as antibacterial food additives.^43^ We also observed uneven and unusual accumulation of RGD-surface-modified bacteriophage on a few enterocytes. A possible explanation for this might be that cells in different phases of the cell cycle can internalize nanoparticles at different rates.^44^

Another significant aspect of a vaccine is its adjuvant ability to enhance the body’s immune response. Adjuvants enhance immunity to vaccines by a variety of mechanisms, namely increased local inflammation, stimulation of non-specific lymphocyte proliferation, and the prolonged persistence of the antigen.^45^ It has been suggested that bacteriophage serves as a strong adjuvant and boosts the immune response; this can be taken advantage of in applications in vaccination.^12^, ^13^ The result of the present study indicates that the engineered bacteriophage has a high potential to induce NO production in macrophages, confirming an adjuvant property of the engineered bacteriophage.

In conclusion, oral vaccination provides both social and economic advantages, especially in developing countries. Despite these promising results, a large range of related parameters needed to be measured and monitored in order to determine induction of antibody and T lymphocyte responses to associated antigens. Further research should be undertaken to investigate the *in vivo* animal studies by using real antigen-producing gene in order to evaluate the effectiveness of the engineered bacteriophage for DNA immunization by oral administration. We expected from this study an efficient, cheap, and safe delivery platform that targets the intestinal cells. This bacteriophage-derived nanovaccine would be a promising platform that may have utility in the targeted oral delivery of DNA vaccines to mucosal immune system within the gastrointestinal tract.

## Materials and Methods

### Materials

The fUSE5-MCS vector and a mammalian DNA cassette containing a GFP gene were kindly gifted from Dr. Amin Hajitou. The HEK293T, the human colonic Caco-2, the human B cell lymphoma (Raji), and the murine macrophage RAW264.7 cell lines were obtained from the American Type Culture Collection (ATCC). Stable HEK293T cells expressing GFP were kindly gifted from Dr. Tawin Iempridee. LB Broth (Miller), tetracycline hydrochloride, rabbit anti-bacteriophage antibody, DAPI, pepsin, pancreatin, and LPS were purchased from Sigma. The DMEM, RPMI 1640 medium, antibiotics, and fetal bovine serum (FBS) were purchased from Gibco. LIVE/DEAD Viability/Cytotoxicity kit, goat anti-rabbit Alexa-Fluor-647-conjugated secondary antibody, and Alexa 488 phalloidin were obtained from Invitrogen. CellTiter-Glo Luminescent Cell Viability assay system and Steady-Glo Luciferase assay system were provided by Promega.

#### Construction and Production of Bacteriophage Vectors

To generate bacteriophage-derived vectors for oral delivery of DNA vaccine, the bacteriophage was genetically engineered to display copies of the RGD peptide, on the pVIII major coat protein, and to carry a mammalian gene cassette encoding a cytomegalovirus promoter-driven transgene expression. Briefly, the RGD peptide-encoding nucleotide was introduced into geneVIII using site-directed mutagenesis, after which the mammalian DNA cassette containing a GFP gene was inserted into the multiple cloning site of the fUSE5-MCS vector. Subsequently, bacteriophage particles were generated and purified from culture supernatants of host bacteria (*Escherichia coli*) by using PEG/NaCl precipitation method as described previously.^17^ Bacteriophage particles were sterile filtered through 0.45-μm filters and then titrated using spectrophotometry method and expressed as mg/mL. Throughout this paper, the abbreviation RGD and NS will be used to refer to the RGD-surface-modified and non-surface-modified bacteriophage, respectively.

### Transmission Electron Microscopy

The RGD-surface-modified bacteriophages were observed by transmission electron microscopy (TEM). For this, 15 μL of each purified phage sample was transferred to a 400-mesh copper grid with a pure carbon film (Electron Microscopy Sciences), incubated for three minutes on the grid, and removed using filter paper. Grids were then washed with two drops of Milli-Q water and dried using filter paper. Then, negative staining with 1% uranyl acetate solution was performed for 30 s, followed by removal of the stain using filter paper, washing with two drops of water, drying, and exposure to osmium vapor a further 1.5 hr. Imaging was performed using a JEOL JEM 1011 transmission electron microscope (JEOL).

#### ζ Potential Measurement

Nanosizer was used for measuring the ζ potential charge of RGD- or NS-bacteriophage in batch mode at 25°C in a quartz microcuvette.

#### Infectivity Assay

The bacteriophage particle number was calculated from measuring protein and DNA by spectrophotometry. An aliquot of bacteriophage solutions being previously adjusted to an identical particle number was incubated with host *E. coli*. The infected cells were plated onto LB plates containing 40 μg/mL tetracycline. Infectivity (colony-forming units/μL) was calculated from the number of colonies that grew on the plates overnight.

### The Stability of Engineered Bacteriophage at Different Acid pH and Enzymatic Fluids

#### Bacteriophage Stability at Different Acid pH

Solutions of RGD-surface-modified bacteriophage were prepared at initial concentrations of 5 mg/mL in 0.2% (wt/vol in water) NaCl adjusted to different pH values (1.0, 2.5, 3.5, 4.5, and 5.7) using hydrochloric acid (pH checked with indicator strips) and incubated at 37°C, with rocking to simulate conditions encountered in the gastrointestinal tract of animals as previously described.^46^ Control with the same conditions but prepared in PBS (pH 7.4) were used. Samples were collected after 1-, 5-, 10-, and 20-min incubations and neutralized with sodium hydroxide and pH checked with indicator strips. Bacteriophages in neutralized samples were immediately assayed for bacteriophage survival. The experiment was replicated three times, and the results are presented in mean survival rate (% of control) + SEM.

#### Bacteriophage Stability in Enzymatic Fluids

The stability of bacteriophage in gastric and pancreatic enzymatic conditions was assessed as previously described.^46^ For this, simulated gastric fluid (SGF) comprised of 3.2 mg/mL pepsin in 0.2% (wt/vol) NaCl at pH 3.5 and simulated intestinal fluid (SIF) comprised of 1 mg/mL pancreatin in 0.2% (wt/vol) NaCl at pH 8.0 were prepared. Bacteriophage were added to the pre-warmed solutions at 37°C at a concentration of 25 mg/mL bacteriophage and incubated at 37°C. Samples were collected at 120 min and immediately assayed for bacteriophage. Controls with the same conditions but without the enzymes were used. The results are presented as mean survival + SEM of the mean of the phages in the enzymatic fluids compared to the respective controls.

#### Cell Culture

HEK293T and Caco-2 cell lines were maintained in complete DMEM supplemented with 10% FBS, penicillin (100 units/mL), streptomycin (100 μg/mL), and L-glutamine (2 mM). RAW264.7 and Raji cells were grown in RPMI 1640 supplemented with 10% FBS, 100 units/mL penicillin, 100 units/mL streptomycin, and 2 mM L-glutamine. Cells were grown at 37°C in a humid atmosphere of 5% CO_2_.

#### *In Vitro* Cytotoxicity

*In vitro* cytotoxicity of bacteriophage was evaluated by CellTiter-Glo Luminescent Cell Viability assay system. Cells were seeded and cultured in medium containing RGD- or NS-bacteriophage with different concentrations. The cytotoxicity of bacteriophage was examined 24 hr post-incubation. Cells were also stained with the reagents in the LIVE/DEAD Cell Viability/Cytotoxicity Assay Kit. Cells were also stained with Alexa-Fluor-584-conjugated phalloidin and DAPI.

#### Immunofluorescence Staining

Cells were incubated with bacteriophage vectors for 4 hr, washed with PBS, and fixed in 4% paraformaldehyde (PFA). After sample preparation by fixation, permeabilization, and blocking, cells were then incubated for 1 hr at room temperature with rabbit anti-bacteriophage antibody. For secondary staining, cells were incubated with goat anti-rabbit Alexa-Fluor-647-conjugated secondary antibodies and with DAPI for 1 hr in darkness at room temperature. Images were obtained using a fluorescent microscope.

#### Cell Transfection and Examination of GFP Gene Expression

All transfection studies were performed in 48-well plates with cells plated 24 hr before transfection at the seeding density of 100,000 cells/mL, 500 μL per well. Bacteriophages (5 mg/mL) were directly added to the cell supernatant and incubated at 37°C. Transfection efficiency as determined by the expression of the GFP reporter gene was assessed using a Nikon Eclipse TE2000-U fluorescence microscope. Photographic images were obtained by using 4× or 10× magnification and fluorescent setting.

### Quantitative Analysis of GFP Level

HEK293T cells were cultivated in 48-well plates to 90% confluency and used for transfection with non-surface-modified and RGD-surface-modified bacteriophage at a tissue culture concentration of 5 mg/mL. Then, transfected cells were incubated at 37°C under a 5% CO_2_ atmosphere for 3 days. For lysis, 100 μL of Glo lysis buffer (Promega) was added to each well and incubated for 10 min at 37°C. Lysates were centrifuged for 5 min at 10,000 *g* in an Eppendorf centrifuge to remove cell debris. One hundred microliters of the supernatants was transferred to an opaque 96-well plate for fluorescence measurement. The fluorescent signal was read in a fluorescence microplate reader with excitation at 488 nm and detection at 507 nm. For competition experiments, 0.2 mg/mL of fibronectin, which has been reported to be an integrin-binding protein,^47^ was added to the wells 30 min before the incubation with bacteriophage. At 3 days post-transfection, the quantity of GFP in sample was determined by comparing its fluorescence reading with that of HEK293T cells stably expressing GFP (virtually 100% stable transfection).

#### *In Vitro* Model of the Human Intestinal Follicle-Associated Epithelium

Caco-2/Raji co-cultivation was established in the Transwell system with a procedure previously described.^48^ Transepithelial electrical resistance (TEER) measurement was also used to assess the formation of a cell monolayer with maintenance of tight junction integrity as previously described.^49^ Only wells with TEER values of at least 0.3 kΩ/cm^2^ were used in permeation experiments. RGD- or NS-bacteriophage diluted in cell culture medium was introduced into the apical chamber of Transwells. The basolateral solution was collected after 4 hr incubation. The number of bacteriophage particles was quantified using the *E. coli* bacterial infection method and counting colony-forming units, as previously described.

#### The Measurement of NO Production

The RAW264.7 cells were pre-incubated with RGD- or NS-bacteriophage for 30 min at 37°C. As a positive control, macrophages were cultured with LPS. Following a 24-hr incubation at 37°C, supernatants were mixed with an equal volume of Greiss reagent and incubated at room temperature for 10 min. Using NaNO_2_ to generate a standard curve, nitrite production was measured by an absorbance reading at 540 nm (A_540_).

#### Statistical Analysis

GraphPad Prism software (version 5.0) was used to perform statistical analyses. Data were presented as mean ± SEM. p values were generated by t test or one-way or two-way ANOVA, considered significant when <0.05, and denoted as follows: *p < 0.05; **p < 0.01; and ***p < 0.001.

## Author Contributions

T.Y. was involved in the design and supervision of the construction and physicochemical characterizations. T.Y., K.N., P.A., and S.B. were involved in conducting physicochemical experiments. T.Y., N.S., S.P., A.H., and K.R. were involved in the design and supervision of the biological assays. T.Y., K.N., S.T., N.N., and M.K. were involved in conducting the biological experiments. T.Y. and S.T. performed the statistical analyses and wrote the manuscript text. All authors reviewed the manuscript.

## Conflicts of Interest

The authors report no conflicts of interest in this work.
